# Cavitation in a soft porous material

**DOI:** 10.1093/pnasnexus/pgac150

**Published:** 2022-08-18

**Authors:** Yu Leng, Pavlos P Vlachos, Ruben Juanes, Hector Gomez

**Affiliations:** School of Mechanical Engineering, Purdue University, 585 Purdue Mall, West Lafayette, IN 47907, USA; School of Mechanical Engineering, Purdue University, 585 Purdue Mall, West Lafayette, IN 47907, USA; Department of Civil and Environmental Engineering, Massachusetts Institute of Technology, 77 Massachusetts Avenue, Cambridge, MA 02139, USA; School of Mechanical Engineering, Purdue University, 585 Purdue Mall, West Lafayette, IN 47907, USA

**Keywords:** cavitation, porous medium, bubble collapse, poroelastic

## Abstract

We study the collapse and expansion of a cavitation bubble in a deformable porous medium. We develop a continuum-scale model that couples compressible fluid flow in the pore network with the elastic response of a solid skeleton. Under the assumption of spherical symmetry, our model can be reduced to an ordinary differential equation that extends the Rayleigh–Plesset equation to bubbles in soft porous media. The extended Rayleigh–Plesset equation reveals that finite-size effects lead to the breakdown of the universal scaling relation between bubble radius and time that holds in the infinite-size limit. Our data indicate that the deformability of the porous medium slows down the collapse and expansion processes, a result with important consequences for wide-ranging phenomena, from drug delivery to spore dispersion.

Significance Statement Cavitation, the liquid–vapor phase transformation of a fluid driven by depressurization, is critical in many science and engineering applications, but it also occurs in our daily lives, for example, when we crack our knuckles or when sea waves break at the beach. Past research has focused on cavitation in free or wall-bounded fluids, but cavitation processes that occur in a soft porous material remain unexplored. Here, we develop a computational model that shows that the deformability of a porous material slows the collapse and expansion of cavitation bubbles and breaks down the classic scaling relation between bubble size and time. Our results have profound consequences for diverse phenomena that involve cavitation in soft porous materials, from traumatic brain injury and drug delivery to spore dispersion.

## Introduction

The collapse of cavitation bubbles in free fluids has fascinated scientists for decades, but what happens when a cavitation bubble collapses in a soft porous material? Cavitation processes are ubiquitous in physics (1,2), engineering (3), and biology (4, 5). They occur in our daily lives when we crack our knuckles and when sea waves break at the beach. Cavitation is also used technologically in medical treatments (6) and cleaning systems (7). These processes involve the nucleation, growth, and collapse of gas bubbles in a liquid. A large body of past work has led to the fundamental understanding of many aspects of cavitation in free liquids (8) and in wall-bounded fluids (9). Recently, there has been increasing interest in understanding the unstable expansion of a bubble in a soft elastic or viscoelastic material (10–13) because it is important for material characterization (14) and it may open opportunities to understand failure in soft solids (15). However, cavitation processes occurring in soft, porous materials, where fluid flow and elasticity interact, have remained unexplored. The energy barrier for nucleation in an elastic porous medium is unknown and we also ignore how much energy is required to expand or collapse a pre-existing gas bubble in an elastic porous medium. These knowledge gaps limit our understanding of critical scientific problems where cavitation occurs in soft porous materials, such as traumatic brain injury (16, 17) and drug delivery (18). In the context of traumatic brain injury, understanding the collapse of cavitation bubbles in poroelastic media may play a critical role in assessing and preventing brain damage. Liquids at conditions similar to those occurring normally in the brain interstitial fluid are known to contain small stable gas bubbles (19,20). If the brain is subjected to a pressure wave, these bubbles will grow when they go through low-pressure conditions. After the growth phase, the bubbles will collapse potentially producing damage in the brain. Having a model of cavitation bubble collapse in poroelastic media is also critical to understand emerging forms of drug delivery for cancer. One example is liposome-assisted drug delivery. In this drug delivery modality, the drug is encapsulated in liposomes, allowing passive accumulation within tumors. The liposomes are designed to remain stable during the delivery phase. The release of the drug at the tumor site is achieved by use of ultrasound waves that produce expansion and subsequent collapse of gas bubbles. The violence of the collapse disrupts the liposome and leads to drug release.

Here, we study the collapse and expansion of a cavitation bubble in a poroelastic material. We formulate a new continuum model that couples compressible fluid flow in the pore network with the elastic response of the solid skeleton. Our theoretical and computational results show that the elasticity of the solid skeleton slows down the collapse process, especially in the final stages. One of the primary driving forces for bubble collapse in a free fluid is the pressure difference between the bubble interior and the far field (3). In a poroelastic material, however, establishing a pressure gradient requires deforming the solid. As a result, pressure gradients are smaller in a poroelastic medium and the collapse is less violent. We also show that the collapse time for a single bubble is predicted by an ordinary differential equation for the bubble radius that extends the Rayleigh–Plesset equation to poroelastic media. The newly proposed equation also shows that the elasticity of the solid skeleton reduces the bubble’s expansion velocity in the presence of ultrasound excitation. Our findings open new opportunities to understand the complex dynamics of cavitation in soft porous materials, which may lead to technological advances in medical imaging (21) and shed light on drug delivery processes that rely on the violent collapse of cavitation bubbles (18,22, 23).

## Proposed model

Our model is based on continuum mechanics and mixture theory (24). In our case of interest, one of the underlying assumptions of the model is that bubbles are much larger than the pore size. To derive our model, we assume small deformation kinematics (25, 26) and adiabatic conditions. The mass conservation equation for the fluid phase is

(1) $$\begin{equation*}
\frac{\partial (\phi _{\rm f}\mathit{ \rho} )}{\partial \mathit{t}} + \nabla \cdot (\mathit{\rho }\boldsymbol{q})=0,
\end{equation*}
$$

where $\phi _{\rm f}(\boldsymbol{x},t)$ is the porosity (volume of pores per unit volume of porous medium) at point $\boldsymbol{x}$ and time *t*, $\rho (\boldsymbol{x},t)$ is the fluid density, and $\boldsymbol{q}(\boldsymbol{x},t)$ is the volumetric flux vector [see (27)]. We model a single-component liquid–vapor system by utilizing a barotropic equation of state similar to that used in refs. (28, 29) for cavitation in free liquids. We assume that the mixture is in local equilibrium and follows an adiabatic path. Our equation of state can be expressed as *p* = *F*(*ρ*), where *p* is the pore pressure and

(2) $$\begin{equation*}
F(\rho )=\mathit{p}_{\rm sat}+ C\left( \frac{1}{\rho _c} - \frac{1}{\rho }\right) + C\, \frac{\mathit{ b}-\rho _c}{\rho _c^2}\, \frac{\rho -\rho _c}{b-\rho }.
\end{equation*}
$$

Here, *p*_sat_ is the saturation pressure, *ρ*_c_ is the density that leads to minimum speed of sound, 2*C*/*ρ*_c_ is the bulk modulus of the fluid at minimum speed of sound, and *b*^−1^ is the fluid’s covolume. The speed of sound predicted by eq. (2) is continuous with respect to *ρ*. For the values of the parameters *p*_sat_, *C, b*, and *ρ*_c_ taken in this paper, eq. (2) produces excellent quantitative agreement with the equation of state employed in ref. (28). The latter, however, leads to a speed of sound that is discontinuous with respect to *ρ*, which introduces challenges in the numerical discretization. We assume that $\boldsymbol{q}$ is given by Darcy’s law,

(3) $$\begin{equation*}
\boldsymbol{q}=-\frac{k}{\mu (\rho )}\nabla p ,
\end{equation*}
$$

where *k* is the absolute permeability and *μ*(*ρ*) is the mixture’s dynamic viscosity. We take *μ*(*ρ*) = *μ*_v_ + (*μ*_l_ − *μ*_v_)(*ρ* − *ρ*_v_)/*b*, where *μ*_l_ and *μ*_v_ represent, respectively, the viscosity of the liquid and gas, and *ρ*_v_ is a representative density of the vapor phase such that *p*_sat_ = *F*(*ρ*_v_). We assume that the collapse process is primarily driven by the difference between a preimposed, far-field static fluid pressure in the liquid phase and the static fluid pressure in the bubble’s interior, thus neglecting any surface tension effects, which can be shown to be small for the cases studied in this work (see the “Supplementary Material” section). Assuming that inertial forces are small, the overall linear momentum balance of the fluid–solid mixture can be written as $\nabla \cdot \boldsymbol{\sigma }+\boldsymbol{b}=\boldsymbol{0}$, where $\boldsymbol{\sigma }$ is the Cauchy stress tensor of the fluid–solid mixture and $\boldsymbol{b}$ represents the body forces per unit volume. In what follows, we neglect gravity and other body forces, which implies $\boldsymbol{b}=\boldsymbol{0}$. Application of the Gibbs–Duhem equation (27) leads to two constitutive equations:

(4) $$\begin{eqnarray*}
\boldsymbol{\sigma } = \boldsymbol{\sigma }_{\rm eff}-\alpha p \boldsymbol{I} ,
\end{eqnarray*}
$$

(5) $$\begin{eqnarray*}
\phi _{\rm f} = \phi _{\rm f}^0 + \alpha \epsilon _{\rm v} + \frac{\alpha -\phi _{\rm f}^0}{K_{\rm s}}p,
\end{eqnarray*}
$$

where $\boldsymbol{\sigma }_{\rm eff}=G(\nabla \boldsymbol{u}+\nabla \boldsymbol{u}^T)+\lambda \nabla \cdot \boldsymbol{u}$ represents the effective stress, $\boldsymbol{u}$ is the displacement field of the solid skeleton, and *G* and λ are the Lamé parameters. In eq. (5), $\phi _{\rm f}^0$ is the porosity of the undeformed configuration, $\epsilon _{\rm v}=\nabla \cdot \boldsymbol{u}$ is the volumetric strain of solid skeleton, and *α* and *K*_s_ are poroelastic properties that characterize the mechanical behavior of the porous solid. In particular, *K*_s_ represents the bulk modulus of the solid grains and *α* is called Biot’s coefficient (27).

Substituting eqs. (2), (3), and (5) into the mass conservation equation for the fluid, and substituting eq. (4) into the linear momentum balance for the fluid–solid mixture, we obtain the governing equations of our model as

(6) $$\begin{eqnarray*}
\!\!\!\alpha \rho \frac{\partial \epsilon _{\rm v}}{\partial t} \!+\! \left[ \frac{W(\rho )}{\mathit{N}} + \phi _{\rm f}^0 \right]\frac{\partial \rho }{\partial t} = \nabla \cdot \left(\frac{kW(\rho )}{\mu (\rho )}\nabla \rho \right),
\end{eqnarray*}
$$

(7) $$\begin{eqnarray*}
\nabla \cdot \boldsymbol{\sigma }_{\rm eff}= \alpha \nabla p,
\end{eqnarray*}
$$

where *W*(*ρ*) = *ρF*^′^(*ρ*) and $1/N=(\alpha -\phi _{\rm f}^0)/K_{\rm s}$. To derive eq. (6), we have assumed that $(\phi _{\rm f}-\phi _{\rm f}^0)/\phi _{\rm f}^0$ is small, a standard approximation in poroelastic models that use small deformation kinematics (27).

## Results

### Collapse of a spherical bubble in a poroelastic medium

**Fig. 1.**
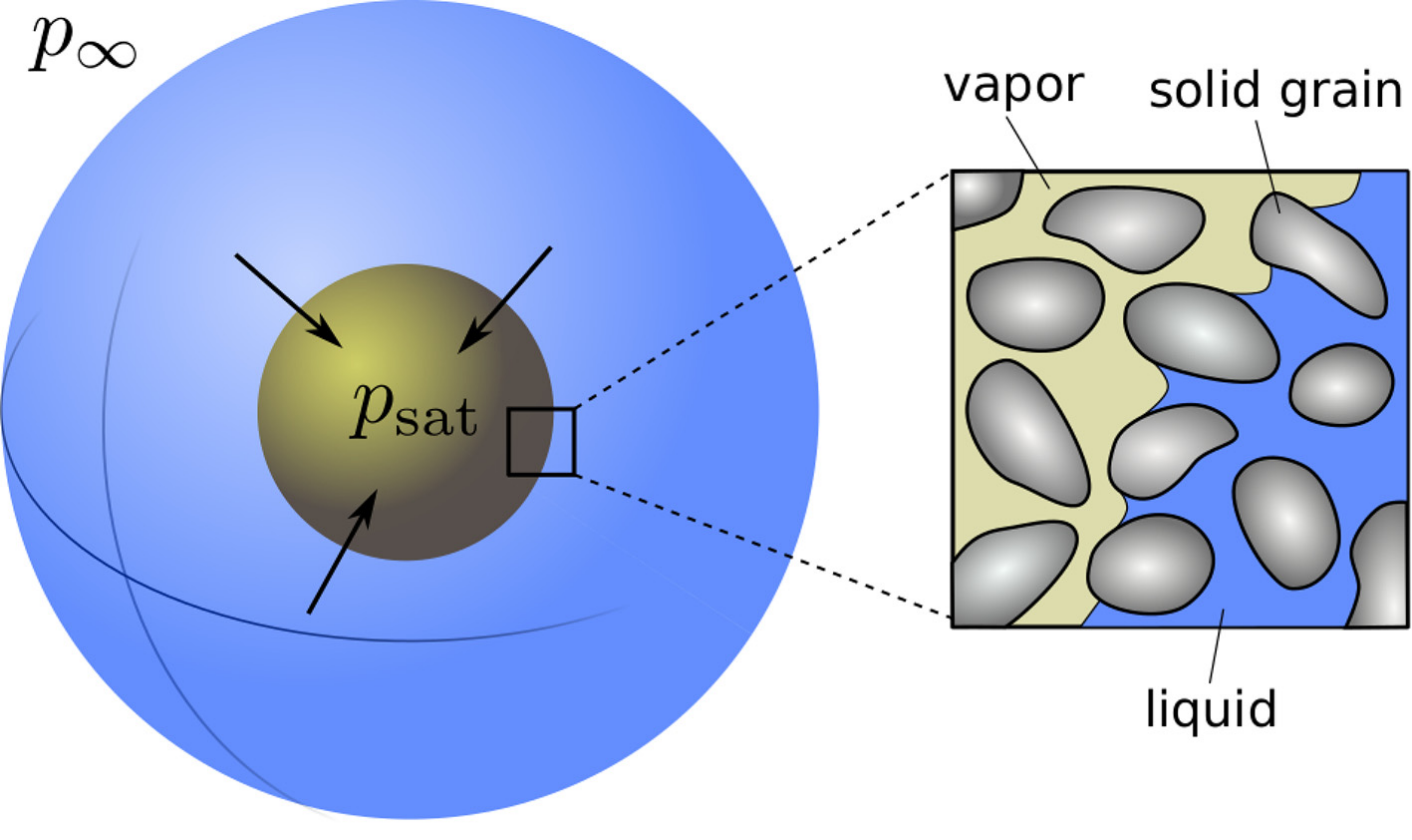
Schematic of a spherical poroelastic medium filled with liquid water (blue) and water vapor (yellow). An overpressure Δ*p* = *p*_∞_ − *p*_sat_ is applied on the external boundary producing the collapse of the bubble, which is accompanied by the deformation of the solid skeleton.

We initially investigate the collapse of a spherical cavitation bubble in a poroelastic medium (see Fig. 1). Under the assumption of spherical symmetry, eqs. (6) and (7) can be simplified to a single scalar equation for the fluid density. By using standard expressions for the differential operators in spherical coordinates, eq. (7) can be written as

(8) $$\begin{equation*}
M\frac{\partial \epsilon _{\rm v}}{\partial r} = \alpha \frac{\partial p}{\partial r},\quad \text{where}\quad \epsilon _{\rm v}=\frac{1}{r^2}\frac{\partial (r^2 u_{\rm r})}{\partial r}.
\end{equation*}
$$

Here, *M* = (*λ* + 2*G*) is the *P*-wave modulus, *r* is the radial coordinate, and *u*_r_ the solid displacement in the radial direction. Integrating eq. (8) leads to *Mϵ*_v_ − *αp* = *g*(*t*). The function *g* can be determined using the boundary conditions. We will focus on the boundary condition *ρ*(*L, t*) = *ρ*_L_, where *L* is the radius of the spherical poroelastic medium and *ρ*_L_ is a given density. Using the boundary condition *ϵ*_v_(*L, t*) = 0 for the linear momentum equation, we obtain *g* = −*αp*_L_, where *p*_L_ = *F*(*ρ*_L_). Taking the time derivative of the expression *Mϵ*_v_ = *α*(*p* − *p*_L_) and substituting in eq. (6), we obtain

(9) $$\begin{equation*}
\left[ B\, W(\rho ) + \phi _{\rm f}^0 \right]\frac{\partial \rho }{\partial t} = \frac{1}{r^2}\frac{\partial }{\partial r} \left(r^2\frac{kW(\rho )}{\mu (\rho )}\frac{\partial \rho }{\partial {\rm r}}\right),
\end{equation*}
$$

where *B* = 1/*N* + *α*^2^/*M*. Once the density field is known, the displacements can be obtained as

(10) $$\begin{equation*}
u_r(r,t)=-\frac{\alpha p_L}{3M}r+\frac{\alpha }{M}\frac{1}{r^2}\int _0^r z^2F(\rho (z,t))\, \mathrm{d} z.
\end{equation*}
$$

For the rest of this work, we parameterize *λ, G*, and *M* using Young’s modulus *E* and Poisson ratio *ν*, namely, *λ* = *Eν*/[(1 + *ν*)(1 − 2*ν*)], *G* = *E*/[2(1 + *ν*)], and *M* = *E*(1 − *ν*)/[(1 + *ν*)(1 − 2*ν*)].

**Fig. 2.**
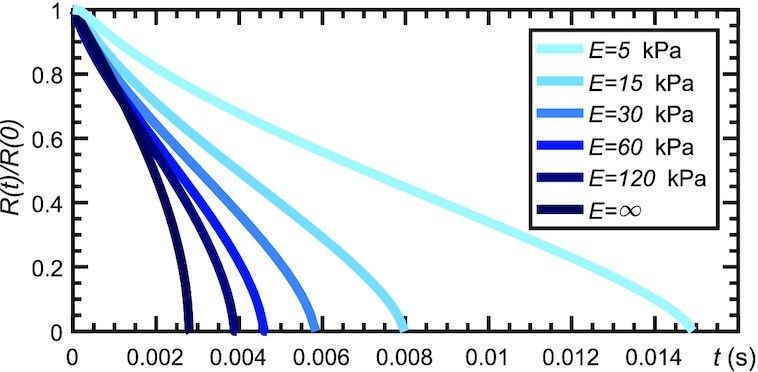
Cavitation bubble collapse in a spherical poroelastic medium. Time evolution of the bubble radius for different values of Young’s modulus. The simulations are performed using isogeometric analysis (30), a generalization of the finite-element method that uses splines of high-order global continuity as basis functions. We used a mesh with 4,096 quadratic elements with globally $\mathcal {C}^1$-continuous basis functions. The time step is selected using the adaptive algorithm described in (31).

We perform numerical simulations of eq. (9) on the domain *r* ∈ (0, *L*), where *L* = 1 mm. The model parameters that are kept fixed for all of the simulations in this paper are shown in Table 1, corresponding to water and a generic soft porous material with properties similar to those of adipose or brain tissue (32,33). Initially, there is a cavitation bubble of radius *L*/2. The density at *r* = *L* is set to *ρ*_L_ = 998 kg m^−^^3^, while the density in the vapor bubble is *ρ* ≈ *ρ*_v_. Figure 2 shows the time evolution of the bubble radius *R*(*t*) for different values of the solid skeleton Young’s modulus. For each value of *E*, we observe a monotonic decrease of the radius with *R* reaching zero at the collapse time. After collapse, the fluid remains in liquid state without any regrowth of the bubble. This was expected and is consistent with results of other models that consider collapse of cavitation bubbles in free liquids without accounting for noncondensable gases (NCGs) (34). The data show that the solid deformation slows down the collapse process. Figure 2 also indicates that, for a rigid porous medium, the velocity of the liquid–vapor interface increases quickly as the bubble shrinks, especially in the final stages of the collapse process. A soft porous medium significantly reduces the acceleration of the interface as evidenced by the curve for *E* = 5 kPa.

**Table 1.** Values of the model parameters.

***α* (−)**	***k* (m^2^)**	***μ*_*l*_ (Pa s)**	***μ*_*g*_ (Pa s)**	***C* (Pa kg m^−3^)**
1	10^−13^	10^−3^	1.3 × 10^−5^	1,450
***p*_sat_ (Pa)**	** $\mathbf{ \phi _{\rm f}^0}$ (−)**	***b* (kg m^−3^)**	**ρ_c_ (kg m^−3^)**	$\mathbf{ K_{\rm s}^{-1}}$ **(Pa^−1^)**
2,339	0.01	998.5	500	0

We assume that the solid grains are incompressible, thus, $K_s^{-1}=0$. This assumption is widely used for soft porous materials (27).

**Fig. 3.**
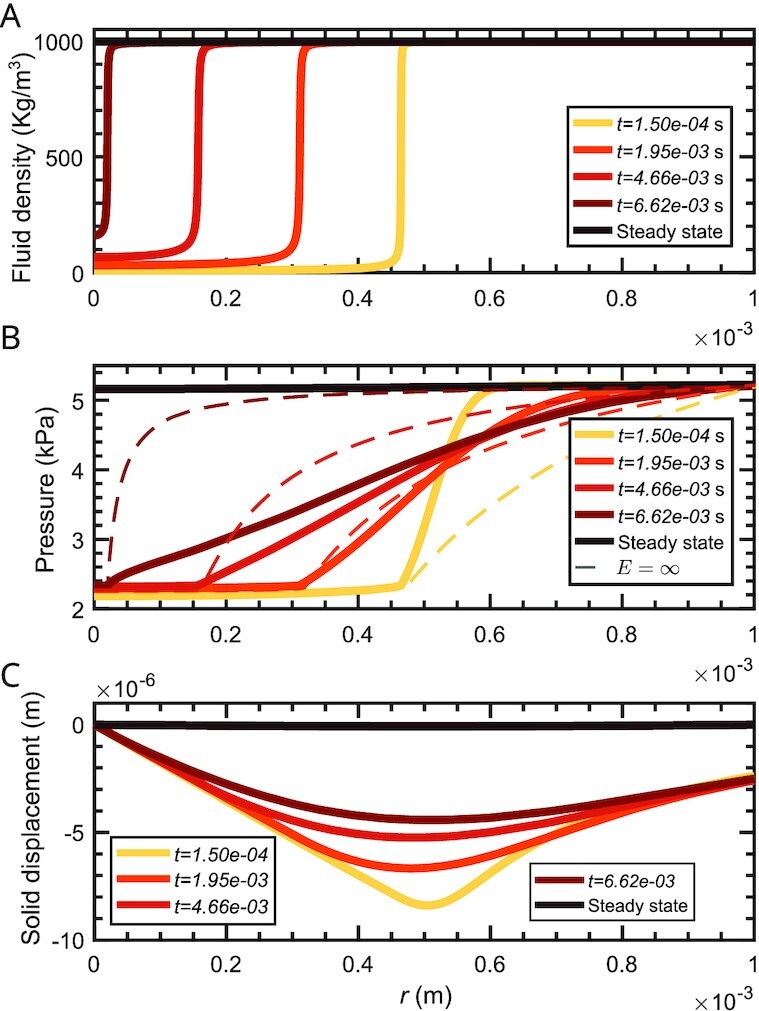
Cavitation bubble collapse in a spherical poroelastic medium. Time evolution of the fluid density (A), pressure (B), and solid displacement (C). We used the parameters given in Table 1 and *E* = 15 kPa, *ν* = 0.45. We repeated this simulation on the domain *r* ∈ (0, 2*L*) with *L* = 1 mm, keeping the same bubble size, and the results (not shown) were nearly identical to those reported here.

Figure 3 shows more details of the collapse process for *E* = 15 kPa. The time evolution of the fluid density is shown in Fig. 3(A). The overpressure at the right boundary produces flow from right to left making the bubble shrink and finally collapse. Figure 3(B) shows that after the liquid–vapor interface starts moving, the pressure in the bubble remains fairly constant, but varies in the liquid phase. The dashed lines in Fig. 3(B) represent the fluid pressure for a similar collapse process in a rigid porous medium. The collapse is faster in a rigid material, so dashed and solid lines of the same color do not correspond to the same time, but rather to times when the bubble radius was the same for the rigid and soft materials. We observe that the pressure gradient at the bubble interface, which is proportional to the interface velocity, is much smaller in the soft material. This can be understood by examining eq. (7), which shows that if a pressure gradient is established in the fluid, the solid needs to be able to produce a counteracting force per unit volume of the same magnitude. This is the primary reason why the collapse is slower in soft materials. Figure 3(C) shows that the solid skeleton is compressed throughout the collapse process and relaxes to an undeformed configuration after the collapse.

### Poroelastic Rayleigh–Plesset equation

We gain further insight into the problem by deriving an ordinary differential equation (ODE) for the time evolution of the bubble radius. The ODE is an extension of the approach used by Rayleigh (35) and Plesset (36) for bubble dynamics in a free liquid. We assume that the solid grains are incompressible (*α* = 1) and that the problem is spherically symmetric. The bubble is centered at *r* = 0, and its radius *R* depends on time. Let us restrict eq. (6) to the domain *r* ∈ (*R, L*), where the fluid is in liquid state and we can assume that the density is approximately constant. Under these assumptions we obtain

(11) $$\begin{equation*}
\frac{\partial \epsilon _{\rm v}}{\partial t} + \frac{1}{r^2}\frac{\partial }{\partial r}\left(r^2q\right)=0,
\end{equation*}
$$

where

(12) $$\begin{equation*}
\epsilon _{\rm v}=\frac{1}{r^2}\frac{\partial (r^2 u_{\rm r})}{\partial r}
\end{equation*}
$$

is the volumetric strain in spherical coordinates. It follows from eq. (11) that

(13) $$\begin{equation*}
r^2(q(r,t)+v_{\rm r}(r,t))=S(t),
\end{equation*}
$$

where *S* is an arbitrary function of time and

$$\begin{equation*}
v_r=\frac{\partial u_r}{\partial t}.
\end{equation*}
$$

If we evaluate eq. (13) at *r* = *R* and identify the fluid velocity at the bubble surface with $\dot{R}$, that is, $q(R,t)=\phi _{\rm f}^0\dot{R}$, we obtain $S=\phi _{\rm f}^0 R^2\dot{R}+R^2v_{\rm r}(R,t)$. Our numerical simulations indicate that the approximation $S\approx \phi _{\rm f}^0 R^2\dot{R}$ leads to a negligible error, so we will use it henceforth. Integrating eq. (13) in the domain (*R, L*), where the fluid density can be assumed to be constant, we have

(14) $$\begin{eqnarray*}
\int _R^L \frac{\phi _{\rm f}^0R^2\dot{R}}{r^2}\, \mathrm{d} r + \int _R^L \frac{k}{\mu }\frac{\partial p}{\partial r}\, \mathrm{d} r = \int _R^L v_{\rm r}(r,t) \, \mathrm{d} r.
\end{eqnarray*}
$$

The left-hand side can be directly integrated under the assumptions of constant permeability and constant viscosity, leading to

(15) $$\begin{equation*}
\phi ^f_0R\dot{R}\frac{L-R}{L} + \frac{k}{\mu } (p_L-p_B) = \int _R^L v_r(r,t) \, \mathrm{d} r ,
\end{equation*}
$$

where we have assumed that *p*(*R*) = *p*_B_. Herein, *p*_B_ is the pressure in the bubble. To transform the right-hand side of eq. (15) into an expression that depends on *R*, we will take the time derivative of eq. (10), which leads to

(16) $$\begin{equation*}
v_r(r,t)=\frac{1}{M}\frac{1}{r^2}\int _0^r z^2F^\prime (\rho (z,t))\frac{\partial \rho }{\partial t}(z,t)\, \mathrm{d} z.
\end{equation*}
$$

From eqs. (15) and (16), we arrive at

(17) $$\begin{eqnarray*}
R\dot{R}\frac{L-R}{L}&=&-\frac{k}{\mu \phi _{\rm f}^0}(p_L-p_B) \nonumber \\
&&+ \ \frac{1}{M\phi _{\rm f}^0}\int _R^L\frac{1}{s^2}\int _0^s \ z^2 \ F^\prime (\rho (z,t))\frac{\partial \rho }{\partial t}(z,t)\, \mathrm{d} z\, \mathrm{d} s.
\end{eqnarray*}
$$

Eq. (17) does not admit closed-form solutions in general. Although eq. (17) was derived by integrating eq. (6) in the liquid phase *r* ∈ (*R, L*), its last term involves values of the density on the entire domain *r* ∈ (0, *s*) with *s* > *R*. Thus, the assumption of constant density that we made for the liquid phase is no longer valid here. Instead, we assume that the density is a traveling wave of the form *ρ*(*z, t*) = *h*(*z* − *R*(*t*)). There are multiple options for the function *h*, but the simplest one is a step function that transitions from *ρ*_v_ to *ρ*_l_ at the bubble surface, i.e. *ρ*(*z, t*) = *ρ*_v_ + (*ρ*_l_ − *ρ*_v_)*H*(*z* − *R*), where *H* is the Heaviside function. This assumption allows approximate integration of the last term in eq. (17) and leads to the equation

(18) $$\begin{equation*}
R\dot{R}(L-R)/L=-\beta ,
\end{equation*}
$$

where

(19) $$\begin{equation*}
\beta = \frac{k(p_L-p_B)/(\phi _{\rm f}^0\mu )}{1+(\rho _l-\rho _v)F^\prime (\rho _R)/(\phi _{\rm f}^0M)}
\end{equation*}
$$

and *ρ*_R_ is the density of the fluid at the bubble interface. Because the fluid density changes abruptly in space at the bubble interface, *ρ*_R_ can be defined in multiple ways. We have determined the value of *ρ*_R_ by fitting one result of the poroelastic Rayleigh–Plesset equation with one high-fidelity simulation and used that value of *ρ*_R_ for all other simulations and all other values of the material parameters.

**Fig. 4.**
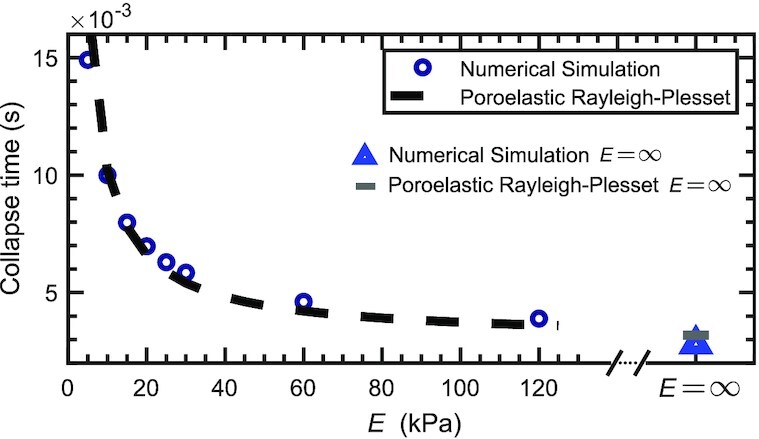
Comparison between the collapse time predicted by the poroelastic Rayleigh–Plesset equation and the collapse time predicted by numerical simulations of eq. (9).

To assess the accuracy of the poroelastic Rayleigh–Plesset, we compare the collapse time predicted by eq. (18) with the collapse time obtained from the full-scale simulations. To perform this comparison, we assume *p*_B_ = *p*_sat_ in eq. (19), which is a common assumption for vapor bubbles without NCG (36). Figure 4 shows good agreement for a large range of Young’s moduli.

The inclusion of *L* as a characteristic length scale in the Rayleigh–Plesset equation plays a critical role in the dynamics of the bubble radius. When this length scale is neglected, i.e. *L* → ∞, the poroelastic Rayleigh–Plesset equation becomes $R\dot{R}=-\beta$. Dimensional analysis shows that the solution to $R\dot{R}=-\beta$ can always be expressed as $\widehat{R}(\hat{t})=\sqrt{1-\hat{t}}$, where $\widehat{R}=R/R(0)$ and $\hat{t}=2\beta t/R(0)^2$. Thus, for *L* → ∞, we can always rescale length and time so that the radius time evolution remains invariant under changes of the model parameters. When *L* < ∞, the universal scaling $\widehat{R}\sim (1-\hat{t})^{1/2}$ does not hold anymore.

### Ultrasonic excitation of a cavitation bubble

**Fig. 5.**
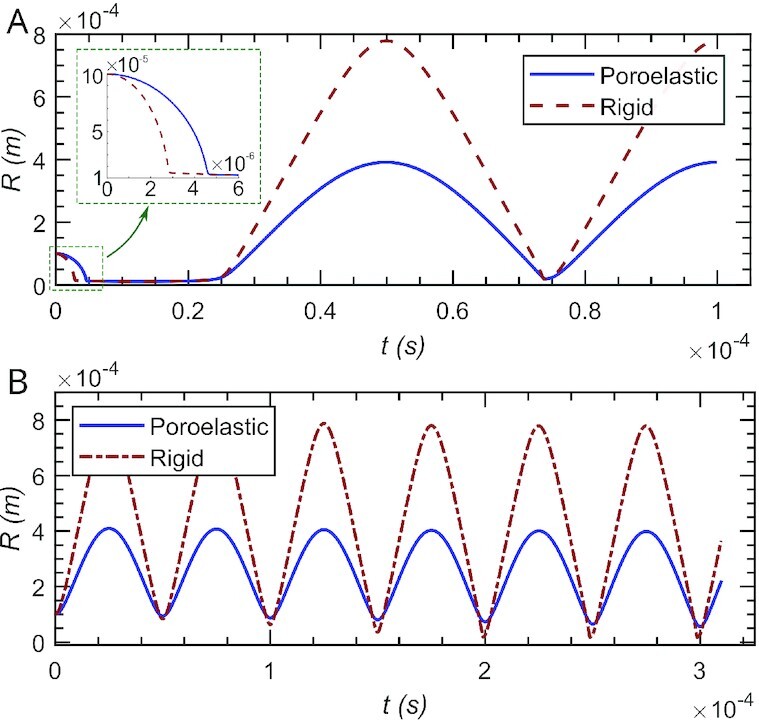
Ultrasonic excitation of a cavitation bubble of initial radius *R*(0) = 100 μm. (A) Time evolution of the bubble radius over two periods of the pressure wave. The bubble initially collapses, and then expands. (B) Time evolution of the radius for a bubble that expands first, and later undergoes six additional cycles of compression and expansion.

We use the poroelastic Rayleigh–Plesset equation to study the dynamics of a bubble under ultrasonic excitation. To avoid singularities in the collapse and to be able to study multiple cycles of expansion and collapse, we assume that, in addition to vapor, the bubble contains an NCG, such as nitrogen. We model this by taking the pressure in the bubble as

(20) $$\begin{equation*}
p_{\rm B} = p_{\rm sat}+ p_{{\rm g}0}\left(\frac{R(0)}{R}\right)^{3\eta },
\end{equation*}
$$

where *p*_g0_ = 0.1 kPa is the NCG pressure for a bubble radius equal to *R*(0). Eq. (20) assumes a polytropic equation of state for the NCG, and that the mass of NCG within the bubble remains constant. If the process is adiabatic, *η* = 1.4 for diatomic gases. We model the ultrasonic excitation by taking the pressure boundary condition as *p*_L_(*t*) = *F*(*ρ*_L_) − ( −1)^*s*^*p*_amp_ sin(2π*ft*), where *p*_amp_ = 1,000 kPa is the ultrasonic wave amplitude, *f* = 20 kHz is the wave frequency, and the constant $s=1,\, 2$ is used to represent two scenarios: when *s* = 1, the bubble collapses first, and then it expands; and when *s* = 2, the bubble expands first, and then it collapses. Figure 5(A) shows the time evolution of the bubble radius over a time span of two periods of the pressure wave for *s* = 1 and *R*(0) = 100 μm. We initially observe a bubble collapse, which is slower in the poroelastic medium (see inset). The bubble remains small and compressed for a time interval, and then it expands. The expansion velocity and the maximum bubble size are smaller in the poroelastic medium. Figure 5(B) shows the time evolution of *R* over six periods of the pressure wave for *s* = 2. In this case, the bubble expands prior to any collapse. The expansion velocity and maximum bubble size are smaller in the poroelastic medium as before. Here, we can also see that the collapse is stronger and the bubble reaches a smaller size in the rigid medium.

### Collapse of a bubble near a rigid wall

**Fig. 6.**
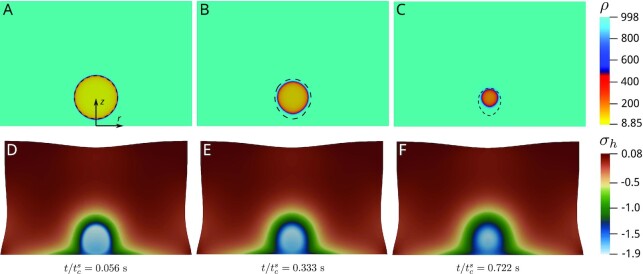
Time evolution of the fluid density *ρ* (kg m^–3^) (A to C) and hydrostatic component of the solid stress *σ*_*h*_ (kPa) (D to F). The snapshots correspond to $t/t_{\rm c}^{\rm s}=$ 0.056 (A and D), 0.333 (B and E), and 0.722 (C and F), where $t_c^s=0.180$ s is the collapse time for a soft porous medium. We used the parameters given in Table 1 and *E*= 5 kPa, *ν* = 0.3. The dashed lines in (A to C) indicate the bubble interface for a similar collapse process in a rigid porous medium (*E* = ∞ kPa) at the same time relative to the collapse time, i.e. $t/t_c^r=$ 0.056, 0.333, and 0.722, where $t_c^r=0.015$ is the collapse time in a rigid porous medium.

In many cases of practical interest, such as drug delivery, cavitation bubbles collapse near a solid surface, instead of in isolation. We study bubble collapse near a solid using axisymmetric simulations in cylindrical coordinates of eqs. (6) and (7). Figure 6(A) shows the density field at the initial time. The initial bubble radius is 1.2 mm and its center is located at 1.5 mm from the bottom boundary, which represents a solid wall. The size of the porous medium shown in the figure is 10 mm × 6.5 mm, and represents a cut-plane of the cylindrical specimen where the cavitation process occurs. On the solid wall, we set the normal component of the Darcy velocity to zero. The density varies between $\sim \,$8.8 kg m^−3^ in the bubble interior to 998.2 kg m^−3^ at the left, right, and top boundaries, where its value is imposed. The solid displacements are set to zero at the bottom boundary. All other boundaries are subject to traction-free conditions. Figure 6(A to C) shows the time evolution of the density in the undeformed configuration. The bubble collapses at time $t_{\rm c}^{\rm s}=0.180$ s (not shown). Figure 6(D to F) shows the hydrostatic stress in the deformed configuration with the displacements magnified by a factor of 2. The solid deformation is slightly larger in the early stages of the collapse process, when the pressure gradient is greatest. Although the overall solid specimen is compressed and shrinks during the collapse process, an analysis of the stress field (see Fig. 6D to F) reveals tensions in the periphery of the computational domain. The dashed lines in Fig. 6(A to C) represent the bubble interface for a similar collapse process in a rigid porous medium (*E* = ∞ kPa) at the same time relative to the collapse time, that is, $t/t_{\rm c}^{\rm r}=0.056$, 0.333, and 0.722, where $t_{\rm c}^{\rm r}=0.015$ s is the collapse time in the rigid porous medium. The comparison shows that for the same time relative to collapse time, the bubble in the rigid medium is larger, which implies that the velocity in the final stages of the collapse is much larger in the rigid medium. For the soft porous medium, not only the collapse is slower, but the collapse point is farther from the wall; both factors contribute to reduce the potential damage to the wall. We observe that the bubble loses its circular shape during the collapse process due to the presence of the solid wall, but this is more evident in the rigid medium.

## Conclusion

In conclusion, cavitation processes occurring in soft porous media had remained unexplored, despite their common occurrence in physics, science, and engineering. The results presented herein show that the collapse and expansion of a cavitation bubble is much slower in a soft porous medium than in a rigid medium. Our model indicates that this occurs because elastic forces reduce the fluid pressure gradients at the bubble interface. The slower collapse has important consequences for technological processes that rely on the violent collapse of cavitation bubbles, such as cavitation-triggered spore dispersion (37) and liposome-assisted drug delivery (18). A relevant extension of this work would be studying how elastic energy stored in the solid during the expansion phase could contribute to accelerate a subsequent collapse. Although our first-order estimates indicate that this effect is small (see the “Supplementary Material” section), a more detailed study is warranted. Future efforts should also include extending the model to the large-deformation regime, studying fracture of the solid skeleton, and understanding the expansion of bubbles smaller than the pore size. We also expect that this research will help address important and outstanding problems such as bubble nucleation in a poroelastic medium.

## Supplementary Material

pgac150_Supplemental_File

## Data Availability

All data needed to evaluate the conclusions of this study are included in the manuscript and Supplementary Material.
