# Supplementary material for: Cavitation in a soft porous material
Source: PNAS Nexus. 2022 Aug 18;1(4):pgac150. doi: 10.1093/pnasnexus/pgac150 (PMC9802157; doi:10.1093/pnasnexus/pgac150)
Supplement: pgac150_Supplemental_File [file pgac150_supplemental_file.pdf]

Supplementary Information for

Cavitation in a soft porous material

Yu Leng, Pavlos P. Vlachos, Ruben Juanes, and Hector Gomez

Hector Gomez
E-mail: hectorgomez@purdue.edu

This PDF file includes:

Supplementary text
SI References

Supporting Information Text

Influence of surface tension. The surface tension at the interface between liquid water and water vapor is $\gamma \approx 70 \cdot 10^{-3}\mathrm{Nm}^{-1}$. Except at the very last part of the collapse process, the bubbles considered in this paper are always larger than one of characteristic radius $R_b = 10^{-4}$ m. The Laplace overpressure at the bubble interior in this hypothetical case would be $\Delta p_{\mathrm{Lap}} = \gamma/R_b = 0.7\mathrm{kPa}$, which is small compared to the overpressure used in the simulations to drive the collapse.

Computational method. For completeness, we briefly introduce the governing equations below and refer readers to the main paper for details of the derivation. The mass and linear momentum conservation equations are given as follows

$$\alpha\rho\frac{\partial\varepsilon_v}{\partial t} + \left[\frac{W(\rho)}{N} + \phi_f^0\right]\frac{\partial\rho}{\partial t} = \nabla\cdot\left(\frac{kW(\rho)}{\mu(\rho)}\nabla\rho\right), \tag{1}$$

$$\nabla\cdot\boldsymbol{\sigma}_{\mathrm{eff}} = \alpha\nabla p. \tag{2}$$

Here, α is Biot's coefficient, ρ is the fluid density, ϵ_v is the volumetric strain, t is time, $W(\rho) = \rho F'(\rho)$ and $p = F(\rho)$, where p is the fluid pressure, represents the equation of state of the fluid. $N = K_s/(\alpha - \phi_0)$ where K_s is the bulk modulus of the solid grains, ϕ_f^0 is the porosity of the porous medium in the undeformed configuration, k is the permeability of the porous medium, $\mu(\rho)$ is the viscosity of the fluid, $\boldsymbol{\sigma}_{\mathrm{eff}}$ is the effective stress tensor of the solid.

Spherically symmetric simulations. Under the assumption of spherical symmetry, the governing equations, Eq. (1) and Eq. (2), can be simplified to a single scalar equation for the density, namely,

$$\left[BW(\rho) + \phi_f^0\right]\frac{\partial\rho}{\partial t} = \frac{1}{r^2}\frac{\partial}{\partial r}\left(r^2\frac{kW(\rho)}{\mu(\rho)}\frac{\partial\rho}{\partial r}\right), \tag{3}$$

where $B = 1/N + \alpha^2/M$, M is the P-wave modulus of the porous medium, and r is the coordinate in the radial direction. We discretize the equation in space using Isogeometric Analysis [1], a generalization of the finite element method with superior approximation properties. The formulation is based on the following weak form of the equation

$$\int_0^L \psi\left[BW(\rho) + \phi_f^0\right]\frac{\partial\rho}{\partial t}\,\mathrm{d}r + \int_0^L \frac{\partial\psi}{\partial r}r^2\frac{kW(\rho)}{\mu(\rho)}\frac{\partial\rho}{\partial r}\,\mathrm{d}r = 0, \tag{4}$$

where L is the length of the porous medium, ψ is a weight function and we have used the boundary conditions to eliminate the boundary terms. We use the Galerkin method which implies that, up to the essential boundary conditions, the discrete spaces for weight and trial functions are identical. Thus, we use the expressions

$$\psi(\boldsymbol{x}) = \sum_A \psi_A N_A(\boldsymbol{x}); \quad \rho(\boldsymbol{x},t) = \sum_A \rho_A(t)N_A(\boldsymbol{x}). \tag{5}$$

Here, the N_A's define a basis of the discrete space, the ψ_A's are constants and the ρ_A's are functions of time. In our computations the N_A's are quadratic splines with continuous derivative across the element boundaries. To linearize the equations, we first write them in residual form. Using the linearity of the weak form with respect to the weight function, we have

$$R_A^S = \int_0^L N_A\left[BW(\rho) + \phi_f^0\right]\frac{\partial\rho}{\partial t}\,\mathrm{d}r + \int_0^L \frac{\partial N_A}{\partial r}r^2\frac{kW(\rho)}{\mu(\rho)}\frac{\partial\rho}{\partial r}\,\mathrm{d}r \text{ for all } A, \tag{6}$$

where R_A^S is the residual. Time integration was performed using the generalized-α method with adaptive time step [2]. The resulting non-linear system of equations was linearized employing Newton's method, and the linear algebraic problem was solved using GMRES [3] with diagonal preconditioner. The code was verified with the MATLAB® PDE solver which is based on the algorithm described in [4].

Cylindrically symmetric simulations. The governing Eqs. (1)-(2) are discretized in space using the finite element method. Our approach is based on the following weak form of Eqs. (5)-(6)

$$0 = \int_\Omega \psi\left\{\alpha\rho\frac{\partial\varepsilon_v}{\partial t} + \left[\frac{W(\rho)}{N} + \phi_f^0\right]\frac{\partial\rho}{\partial t}\right\}\mathrm{d}\Omega + \int_\Omega \frac{kW(\rho)}{\mu(\rho)}\nabla\psi\cdot\nabla\rho\mathrm{d}\Omega - \int_\Gamma \psi\frac{kW(\rho)}{\mu(\rho)}\nabla\rho\cdot\boldsymbol{n}\mathrm{d}\Gamma, \tag{7}$$

$$0 = \int_\Omega \nabla\boldsymbol{\omega} : \boldsymbol{\sigma}_{\mathrm{eff}}\mathrm{d}\Omega + \int_\Omega \boldsymbol{\omega}\cdot\alpha\nabla p\;\mathrm{d}\Omega - \int_\Gamma \boldsymbol{\omega}\cdot\boldsymbol{\sigma}_{\mathrm{eff}}\boldsymbol{n}\mathrm{d}\Gamma, \tag{8}$$

where Γ is the Neumann boundary of the domain of interest Ω and $\boldsymbol{n}$ is the unit outward vector normal to Γ. We formulate the problem using the density ρ and the displacements $\boldsymbol{u}$ as unknowns. We use the Galerkin method. Thus, the density, displacements, and the weight functions ψ and $\boldsymbol{\omega}$ can expressed as in Eq. (5). For cylindrically symmetric simulations, however, the N_A's are piecewise linear functions defined over a triangulation of the computational domain because this facilitates local

mesh refinement. Using the boundary conditions and the linearity of the weak form with respect to the weight function, Eqs. (7)-(8) can be written in residual form as

$$R_A^m = \int_\Omega rN_A\left\{\alpha\rho\frac{\partial\varepsilon_v}{\partial t} + \left[\frac{W(\rho)}{N} + \phi_f^0\right]\frac{\partial\rho}{\partial t}\right\}\mathrm{d}\Omega + \int_\Omega \frac{\partial N_A}{\partial r}\frac{kW(\rho)}{\mu(\rho)}r\frac{\partial\rho}{\partial r}\mathrm{d}\Omega + \int_\Omega \frac{\partial N_A}{\partial z}\frac{kW(\rho)}{\mu(\rho)}r\frac{\partial\rho}{\partial z}\mathrm{d}\Omega, \tag{9}$$

$$R_{Ar}^M = \int_\Omega \frac{\partial N_A}{\partial r}\sigma_{rr} + \frac{\partial N_A}{\partial z}\sigma_{rz}\mathrm{d}\Omega + \int_\Omega N_A\frac{\sigma_{rr}}{r}\mathrm{d}\Omega + \int_\Omega N_A\alpha\frac{\partial p}{\partial r}\mathrm{d}\Omega, \tag{10}$$

$$R_{Az}^M = \int_\Omega \frac{\partial N_A}{\partial r}\sigma_{rz} + \frac{\partial N_A}{\partial z}\sigma_{zz}\mathrm{d}\Omega + \int_\Omega N_A\frac{\sigma_{rz}}{r}\mathrm{d}\Omega + \int_\Omega N_A\alpha\frac{\partial p}{\partial z}\mathrm{d}\Omega, \tag{11}$$

where R_A^m, R_{Ar}^M and R_{Az}^M are residuals, r and z are cylindrical coordinates on Ω, the volumetric strain ε_v in the coordinate system is given as

$$\varepsilon_v = \frac{\partial u_r}{\partial r} + \frac{u_r}{r} + \frac{\partial u_z}{\partial z}, \tag{12}$$

and stress components $\sigma_{rr}, \sigma_{zz}, \sigma_{rz}$, and σ_{zr} become

$$\sigma_{rr} = \lambda\left(\frac{1}{r}\frac{\partial(ru_r)}{\partial r} + \frac{\partial u_z}{\partial z}\right) + 2G\frac{\partial u_r}{\partial r}, \quad \sigma_{zz} = \lambda\left(\frac{1}{r}\frac{\partial(ru_r)}{\partial r} + \frac{\partial u_z}{\partial z}\right) + 2G\frac{\partial u_z}{\partial z}, \quad \sigma_{rz} = \sigma_{zr} = G\left(\frac{\partial u_z}{\partial r} + \frac{\partial u_r}{\partial z}\right). \tag{13}$$

Herein, λ and G are the Lamé coefficients, and u_r and u_z are the r and z components of the displacement field.

Time integration is performed using Backward Differentiation Formulas [5] of adaptive order and time step size. The system is solved monolithically. The residuals are linearized using Newton's method and the resulting linear systems are solved by the multifrontal method MUMPS [6]. The algorithm was implemented in COMSOL Multiphysics® through a Weak Form Interface. The mesh was locally refined with smaller elements in the collapse region.

Influence of the stored elastic energy. Our manuscript shows that when a bubble is expanded by ultrasonic excitation in a poroelastic medium, the solid is stretched. The elastic energy stored during the expansion phase could contribute to a faster collapse. Here, we evaluate this effect. In the current model, as described by Eq. (1)–Eq. (2), even if the solid is initially stretched, the results will not vary. This is a consequence of our assumption of negligible inertial forces. According to Eq. (2), the force balance is established instantaneously and the initial displacements do not play a role.

We present below an estimate which indicates that, at least for the problems studied in this paper, the effect of stored elastic energy is small. Let's assume that the solid is initially stretched. That will impact flow in two primary ways: (a) Because the solid is expanded, the porosity will increase, and the fluid velocity will be smaller. This would actually slow down the collapse, so we will ignore this effect. (b) The first term on the left hand side of Eq. (1) will contribute to a faster collapse. We estimate the impact of this term in the collapse process. We obtain our estimate by ignoring the right hand side of Eq. (1) and finding an approximate solution to the remaining equation. The effect of the right-hand side of Eq. (1) towards collapse would be added on top, but we want to focus on the contribution of the initial stretch. For simplicity, we proceed in a flat one-dimensional space. Thus, for incompressible solid grains,

$$\frac{1}{\rho}\frac{\partial\rho}{\partial t} = -\frac{\alpha}{\phi_f^0}\frac{\partial u}{\partial x\partial t} \tag{14}$$

where u is the solid displacement. We follow a procedure that is analogous to that used to derive the poroelastic Rayleigh-Plesset equation. We first integrate on the domain $x \in (0, L)$ to obtain

$$\int_0^L \frac{1}{\rho}\frac{\partial\rho}{\partial t}\mathrm{d}x = -\frac{\alpha}{\phi_f^0}\frac{\mathrm{d}u(L,t)}{\mathrm{d}t} \tag{15}$$

where we have assumed that the left boundary is fixed, i.e., $u(0,t) = 0$. The initial stretch will relax to zero in a time t_r that we call recovery time. We now make the approximation $\mathrm{d}u(L,t)/\mathrm{d}t \sim -u(L,0)/t_r$. As done in the derivation of the poroelastic Rayleigh-Plesset equation, we assume that the density is a traveling wave of the form $\rho(z,t) = \rho_v + (\rho_l - \rho_v)H(z - R)$, where H is a Heaviside function. This leads to

$$\dot{R}t_r = -\frac{\alpha}{\phi_f^0}\frac{\rho_R}{\rho_l - \rho_v}u(L,0) \tag{16}$$

where ρ_R is defined as in the manuscript. Assuming that the bubble expansion produces a stretch that is similar to the compression produced by the collapse shown in the simulation presented in fig. 3 of the main text, we obtain $\dot{R}t_r \approx -0.024R(0)$. This implies that, in this case, the initial stretch of the solid would contribute to a reduction of the bubble radius equivalent to $\sim$2.4% of the initial radius.

References

1. TJ Hughes, JA Cottrell, Y Bazilevs, Isogeometric analysis: Cad, finite elements, nurbs, exact geometry and mesh refinement. *Comput. Methods Appl. Mech. Eng.* **194**, 4135–4195 (2005).
2. H Gómez, VM Calo, Y Bazilevs, TJ Hughes, Isogeometric analysis of the cahn–hilliard phase-field model. *Comput. Methods Appl. Mech. Eng.* **197**, 4333–4352 (2008).
3. Y Saad, MH Schultz, Gmres: A generalized minimal residual algorithm for solving nonsymmetric linear systems. *SIAM J. on scientific statistical computing* **7**, 856–869 (1986).
4. RD Skeel, M Berzins, A method for the spatial discretization of parabolic equations in one space variable. *SIAM journal on scientific statistical computing* **11**, 1–32 (1990).
5. PR Amestoy, IS Duff, JY L'excellent, Multifrontal parallel distributed symmetric and unsymmetric solvers. *Comput. methods applied mechanics engineering* **184**, 501–520 (2000).
6. MS Plesset, A Prosperetti, Bubble dynamics and cavitation. *Annu. Rev. Fluid Mech.* **9**, 145–185 (1977).
